# Quantitative ex vivo assessment of target temperature and ablation duration for protocol optimization of microwave ablation procedures with mr thermometry

**DOI:** 10.1038/s41598-026-41656-3

**Published:** 2026-03-03

**Authors:** Luigi Nardone, Alexander Sheng Ming Tan, Pierre Bour, Matthias Philipp Fabritius, Elif Öcal, Vanessa Franziska Schmidt, Mingming Wu, Laura Maria Bauer, Valéry Ozenne, Jens Ricke, Max Seidensticker, Olaf Dietrich

**Affiliations:** 1 Department of Radiology, LMU University Hospital, LMU Munich, Marchioninistr. 15, 81377 Munich, Germany; 2 Institute of Radiology, Department of Medicine, University of Udine, Udine, Italy; 3 Department of Vascular and Interventional Radiology, Singapore General Hospital, SingHealth Duke-NUS Radiological Sciences Academic Clinical Programme, Singapore, Singapore; 4 Certis Therapeutics, Pessac, France; 5 Univ. Bordeaux, CNRS, CRMSB, UMR 5536, IHU Liryc, Bordeaux, France

**Keywords:** MRI-guided ablation, MRI thermometry, Interventional radiology, Image quality assessment, Microwave ablation liver, Engineering, Medical research

## Abstract

To quantitatively assess the influence of target temperature and ablation duration on the quality of proton resonance frequency shift (PRFS)-based MR thermometry during microwave ablation (MWA) in a controlled ex vivo model, and to identify parameter ranges associated with improved thermometry performance. Thirty-two MWAs were performed in 10 ex vivo bovine livers in a 1.5-tesla MRI system with multi-slice volumetric real-time thermometry yielding temperature and thermal dose maps. The experiments were conducted twice using all combinations of four target temperatures (60; 80; 100; 120 °C) and four ablation times (5:00; 7:30; 10:00; 15:00 min). Thermometry quality was rated on a 5‑point Likert scale. Ablation areas were compared with histopathology (hematoxylin and eosin, H&E; and nicotinamide adenine dinucleotide, NADH‑diaphorase) and correlated using Spearman coefficients. Likert scores were compared across temperatures using Kruskal-Wallis and Mann-Whitney U tests. All evaluations were performed independently by two readers. Lesion areas varied from 2.6 to 12.9 cm², increasing primarily with target temperature. Ablation areas from temperature and thermal dose maps correlated strongly with macroscopically visual necrosis (*p* < 0.01). Likert scores differed significantly across temperatures (*p* < 0.05). The highest image quality was achieved at 60 °C for 7:30 min, showing comparable scores as at 80° for 15:00 min, but significantly differing from 100 °C to 120 °C. In this controlled ex vivo setting, lower target temperatures were associated with improved MRI thermometry quality, providing more reliable visualization of ablation zones; however, ablation volumes decreased at lower temperatures. Furthermore, these empirical ex vivo observations suggest that a staged two-level approach may support a clinical workflow strategy aimed at balancing thermometry image quality and ablation volume. Given the absence of perfusion and motion effects, these findings require further validation before clinical translation.

**Supplementary Information:**

The online version contains supplementary material available at 10.1038/s41598-026-41656-3.

## Introduction

Microwave ablation (MWA) has emerged as an important therapeutic option for patients with hepatocellular carcinoma or selected liver metastases who are unsuitable for surgical resection, offering promising local control with minimal invasiveness and contributing to improved disease-free survival^1–6^. Compared to radiofrequency ablation (RFA), MWA provides faster and more uniform heating, achieving higher complete ablation rates, lower local progression, and reduced recurrence^7–9^. Advantages of MWA include higher thermal efficiency and reduced susceptibility to heat sink effects from adjacent vessels^10,11^.

Accurate imaging guidance is crucial for safe and effective MWA. Ultrasound (US) is widely used, but its effectiveness is limited by poor contrast, gas interference, and bubble formation during ablation^12,13^. Computed tomography (CT) provides accurate imaging, though only briefly after intravenous contrast administration, and is affected by needle-related artifacts that limit detection of small lesions (≤ 1 cm)^12–17^.

Magnetic resonance imaging (MRI) is increasingly applied to overcome these limitations^18^. MRI offers high soft-tissue contrast, excellent visualization of small lesions (< 1 cm) after liver-specific contrast administration^19,20^. Uniquely, MRI enables quantitative thermometry through the linear proton resonance frequency (PRFS) shift, providing real-time assessment of tissue temperature and predicting the extension of the ablation zone based on the applied thermal dose^21–24^. This facilitates the detection of minimal ablative margins, potentially reducing local tumor progression and improving treatment success^25^.

Nevertheless, MRI-guided MWA remains challenged by magnetic susceptibility effects, signal loss, geometric distortions, and motion artifacts, all of which reduce the quality of thermometry maps^26^. Nonlinear heating effects, including tissue vaporization, represent a major source of error by distorting calculated temperatures^27–29^. Addressing such artifacts through optimized heating protocols is essential for accurate intra-procedural monitoring. Despite advances in MR thermometry for image-guided ablation, the influence of heating profiles and target temperature settings on thermometric accuracy and visualization quality remains poorly quantified. Understanding these dependencies is critical for establishing reference conditions and improving the reliability of real-time MR thermal monitoring. We hypothesized that lower target temperatures could improve MR thermometry reliability by minimizing susceptibility and nonlinear heating effects. The present study therefore aimed to optimize MRI thermometry quality during MWA in an ex vivo animal liver model by systematically varying target temperatures (60–120 °C) and ablation times (5:00–15:00 min) and comparing thermometry visualization quality and reliability between these settings.

## Materials and methods

### A Liver specimens

In total, 10 bovine livers ex vivo were used within 5 h postmortem to minimize autolytic alterations and ensure reliable results^30^. Bovine liver specimens were obtained from a licensed retail butcher shop routinely providing tissues for the human food supply. At the request of the investigators, the animals were slaughtered earlier the same morning, approximately 2 h before sample collection. No animals were euthanized specifically for this study. The liver was first stored in a cold-storage room and then subsequently allowed to reach room temperature in the MR suite. Immediately prior to ablation, tissue temperature was measured using the thermometer integrated into the microwave applicator needle, indicating baseline values of approximately 18 °C.

### B MR-guided MWA setup

An MR-compatible MWA system (MedWaves Avecure, Medwaves, San Diego, CA, USA) with a microwave power of 40 W at frequencies of 902 to 928 MHz was used, which was placed within the MR room. A 14-gauge microwave antenna (AveCure 14 Gauge Probe Medium Antenna, MedWaves, San Diego, CA, USA) without active needle cooling (usable length: 14 cm; diameter: 2 mm) was inserted approximately horizontally (from right to left) into the liver, oriented perpendicular to the main magnetic field (*B*₀).

Ablations were performed in temperature‑control mode with pulsed output power, while the generator frequency was automatically adjusted. Each of the 16 parameter combinations (target temperatures: 60, 80, 100, 120 °C; ablation times: 5:00, 7:30, 10:00, 15:00 min) was repeated once, yielding a total of 2 × 4 × 4 = 32 ablations.

### C Imaging setup

All procedures were performed at a 1.5 T whole-body MRI system (Magnetom SolaFit, Siemens Healthineers, Erlangen, Germany) with a closed-bore design (inner bore diameter: 70 cm). The built-in spine coil array was used for signal reception. The imaging protocol began with T1-weighted 3D gradient-echo acquisitions (axial and coronal) to plan needle placement.

Needle positioning was guided by a real‑time T1‑weighted gradient‑echo sequence in axial, coronal, and sagittal planes, enabling the avoidance of large vessels that could affect ablation. Real‑time images were displayed on an MR‑compatible monitor within the MR room. After needle placement, T1‑weighted 3D gradient‑echo acquisitions were repeated (axial and coronal) to confirm positioning and prepare the ablation sequence.

For MRI thermometry, a segmented multi-slice 2D gradient‑echo echo planar imaging (EPI) sequence (TE 19 ms, TR 47 ms, flip angle 40°, echo-train length 13, parallel imaging acceleration *R* = 2) was used during ablation, providing volumetric coverage through multiple parallel slices with inter-slice gaps. Slices were oriented (par)axially, aligned with the microwave antenna. The spatial resolution was 2.3 × 2.3 mm², slice thickness 3 mm, with 1.5 mm gaps between 13 slices; temporal resolution was 3.6 s. Baseline monitoring was performed for 1 min prior to ablation, followed by 2 min post‑ablation to track temperature decay.

**Fig. 1:**
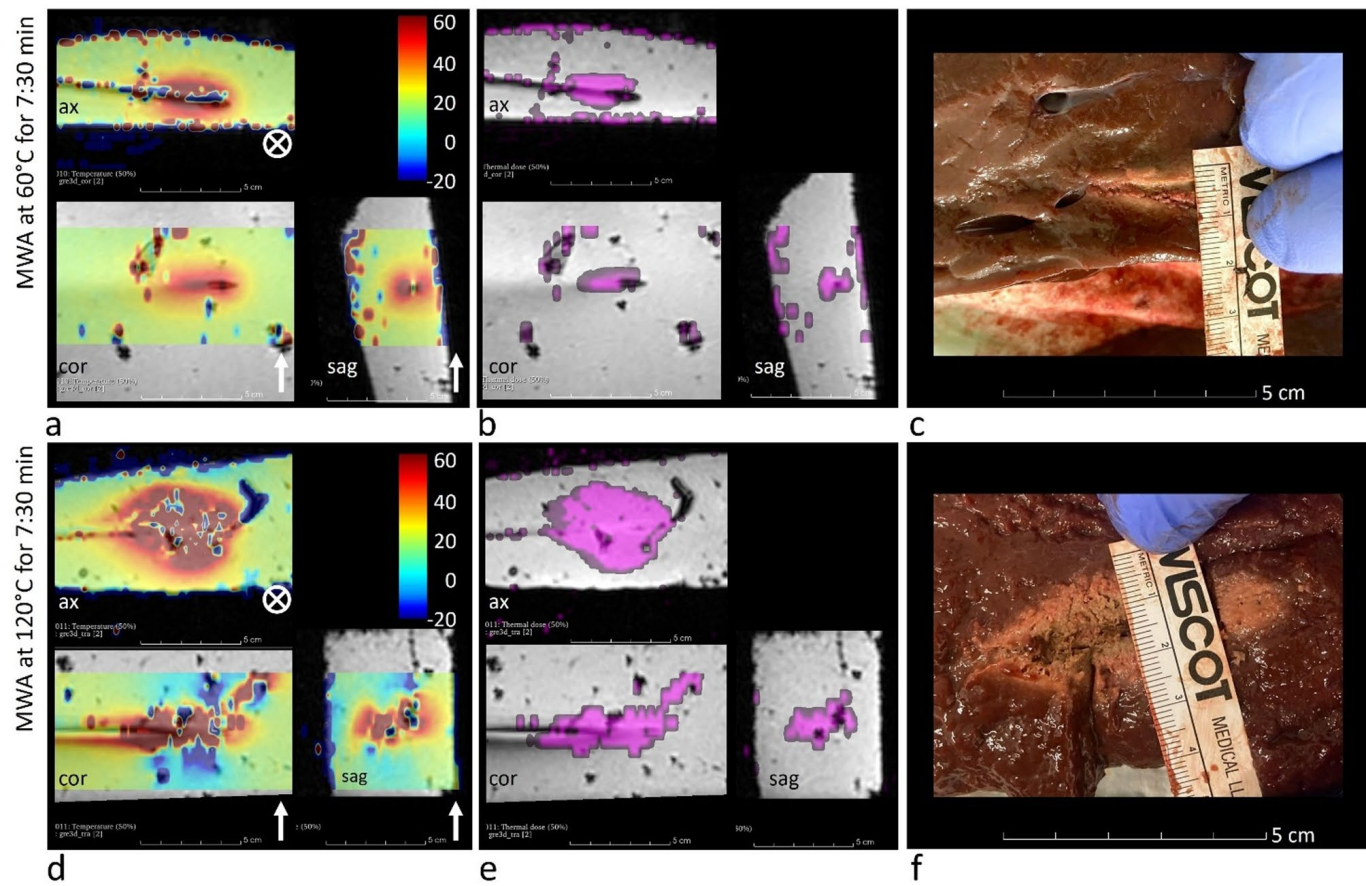
Experimental setup. Experimental setup of two MWAs performed at 60 °C (a, b,c) and 120 °C (d, e,f) with temperature maps (a, d), thermal-dose maps (b, e), and assessment of the macroscopically visual lesion size (c, f). Note the lower map quality at 120 °C along the *B*_0_ field direction (d, e; bottom views, *B*_0_ direction is indicated by the white arrows and circle symbols). Apparently decreasing or even negative temperature values (blue regions) reflect susceptibility-induced PRFS artifacts related to tissue vaporization and gas formation near the antenna, rather than true temperature decreases. All thermometry maps are shown in planes perpendicular to the applicator axis, which was approximately horizontal from right to left. The orientations of the imaging planes (axial, coronal, sagittal) are explicitly indicated. Note the different scaling/magnification in (a, b,d, e) vs. (c, f).

Thermometry maps (temperature and thermal dose) were calculated on an external workstation with a dedicated software package (Certis Solutions, Certis Therapeutics, Pessac, France), see Fig. 1a, b,d, e. The thermometry pipeline includes an image-based temperature drift correction and noise suppression implemented by the Certis platform, in addition to real-time temperature and thermal dose calculation.

The cumulative thermal dose was calculated using the Sapareto–Dewey equation, relating temperature rise, exposure time, and cell death^31^. The lesion extent was estimated at the lethal threshold of 240 min at 43 °C (cumulative equivalent minutes, CEM43). The 240 CEM43 contour was used as a conventional reference boundary to facilitate standardized comparisons across protocols, rather than as an absolute lethality threshold for room-temperature ex vivo tissue.

### D Evaluation of ablation areas

To quantitatively evaluate the MWA performance at the 16 temperature/time settings and to compare these results to MRI thermometry, ablation size was determined as two‑dimensional areas on both liver tissue sections and MRI thermometry maps.

Measurements were performed in planes covering the full antenna length by recording short (perpendicular) and long (parallel) axes of the ablation zone. Liver sections were cut para-axially along the needle, with treated areas identified by color and texture changes (Fig. 1c, f). The same slice was used to assess axes on temperature and thermal-dose maps. On temperature maps, ablation extent was defined as regions reaching ≥ 60 °C at the end of heating, following prior literature^32,33^. Ablation area was calculated as an ellipse (*A* = π × *a* × *b*, where *a* and *b* are semi-axes). Thus, three comparable ablation areas were obtained from (1) tissue sections (i.e., the reference area of actual visual necrosis), (2) temperature maps, and (3) thermal-dose maps.

The evaluation of the ablation area was performed by two radiologists trained in MWA under MRI guidance.

### E Evaluation of MRI thermometry maps (lesion roundness and Likert scale)

To assess thermometry map quality, we evaluated lesion roundness and assigned a 5‑point Likert score reflecting both ablation shape and imaging artifacts (needle and RF artifacts). The roundness index (RI) was defined as the ratio of long to short axes on para-sagittal thermometry maps perpendicular to the needle **(**Fig. 1a, b,d, e; **bottom right view)**.

The Likert scale was designed to reflect predefined combinations of lesion geometry and artifact burden, integrating both ablation shape and imaging artifacts (needle- and RF-related).

The Likert scale (from lowest to highest image quality) was based on the criteria:

1. Many artifacts obscuring evaluation; markedly non-circular lesion (RI ≥ 1.6).
2. Artifacts near needle affecting evaluation; oval lesion (RI < 1.6 but ≥ 1.4).
3. Artifacts within liver but not near needle; oval lesion (RI < 1.4 but ≥ 1.2).
4. Background artifacts without impact; nearly round lesion (RI < 1.2 but ≥ 1.1).
5. Artifact-free; round lesion (RI ≈ 1).

The evaluation of the map quality was performed by two radiologists trained in MWA under MRI guidance. The two readers had different professional backgrounds and were affiliated with independent institutions, reflecting heterogeneous clinical and training environments.

In cases of significant discrepancy between the RI and the level of artifacts, an average of the two scores was used in the Likert score. The final decision on the score was determined by the expert readers, based on their experience and professional judgment.

### F Histological sample analysis

Histological sampling was performed within a consistent time window (30–60 min after ablation) to allow thermal equilibration and to reduce the impact of transient post-heating expansion of necrosis. A perpendicular tissue section was obtained. Samples from the ablation center (including margins and adjacent viable tissue) were fixed in 4% formaldehyde. A second sample was frozen in liquid nitrogen at − 80 °C for further analysis^34,35^. Hematoxylin and eosin (H&E) and NADH-diaphorase staining were performed to assess cell vitality (see Supplementary Material).

### G Statistical analysis

Analyses were conducted using SPSS Statistics (Version 29.0.2.0; IBM, Armonk, NY, USA). Data are reported as median (range). Two-way ANOVA tested effects of temperature and ablation time on lesion area. Correlations of temperature and thermal-dose map areas with necrosis (tissue reference) were assessed using Spearman coefficients, linear regression, and Bland–Altman analysis. Likert scores (ordinal) were compared across temperatures using the Kruskal-Wallis test and the Mann-Whitney U test. Inter-reader agreement for necrosis area measurements was evaluated with the intraclass-correlation coefficient (ICC). For all evaluations, both repeated measurements were analysed. Significance was defined as *p* < 0.05 (two-sided).

## Results

### Quantification of ablation area

**Fig. 2:**
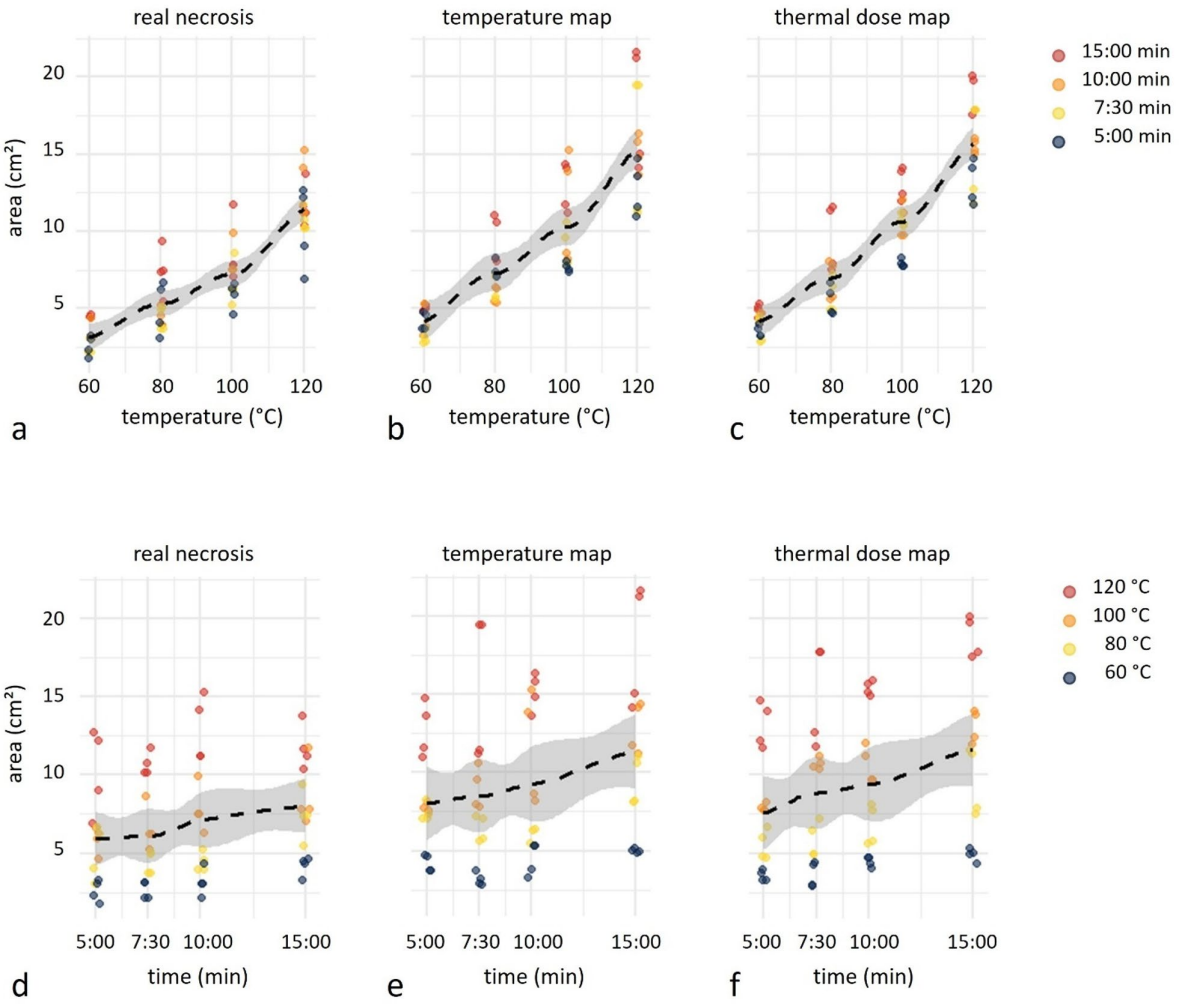
Area Distribution. Distribution of measured areas (cm²) derived from real necrosis (a, d), temperature maps (b, e), and thermal-dose maps (c, f). Each parameter combination includes two independent measurements per reader. Top row (a, b, c): area distribution by temperature, with points coloured according to microwave-ablation duration (5:00 min = dark blue; 7:30 min = yellow; 10:00 min = orange; 15:00 min = red). Bottom row (d, e, f): area distribution by MWA duration, with points coloured according to temperature category (60 °C = dark blue; 80 °C = yellow; 100 °C = orange; 120 °C = red). Dashed black lines represent LOESS regression fits, with shaded bands indicating 95% confidence intervals.

Macroscopically assessed lesion cross-section areas in the central paraxial slice after MWA ranged from 2.6 to 12.9 cm², varying with target temperature (60 °C to 120 °C) and, to a lesser extent, with ablation duration (5:00–15:00 min) (Fig. 2, left column); detailed values are reported in Table 1 (top rows).

**Table 1:** Lesion areas in tissue and MRI thermometry maps. Lesion areas in tissue sections (“real necrosis”) and MRI thermometry maps. Values in the table represent the mean of the measurements obtained by the two readers.

	Temperature	60 °C		80 °C		100 °C		120 °C	
	MWA duration	Long × short diameter (cm × cm)	Area (cm²)	Long × short diameter (cm × cm)	Area (cm²)	Long × short diameter (cm × cm)	Area (cm²)	Long × short diameter (cm × cm)	Area (cm²)
Real necrosis area	5:00 min	2.9 × 1.1	2.6	4.4 × 1.4	5.0	4.2 × 1.7	5.9	5.6 × 2.0	9.0
	7:30 min	3.7 × 0.9	2.6	4.3 × 1.2	4.3	4.8 × 1.7	6.6	5.1 × 2.7	10.7
	10:00 min	3.8 × 1.0	3.2	4.3 × 1.3	4.4	4.9 × 2.0	7.8	5.8 × 2.8	12.9
	15:00 min	3.6 × 1.5	4.2	4.2 × 2.3	7.4	4.8 × 2.2	8.6	5.7 × 2.6	11.8
Temperature map area	5:00 min	3.5 × 1.5	4.2	4.5 × 2.1	7.4	4.5 × 2.2	7.7	5.7 × 2.8	12.7
	7:30 min	3.5 × 1.1	3.1	4.5 × 1.8	6.4	5.1 × 2.2	9.0	5.9 × 3.3	15.4
	10:00 min	3.6 × 1.5	4.4	4.2 × 1.8	5.9	5.6 × 2.6	11.5	6.2 × 3.1	15.1
	15:00 min	3.7 × 1.7	5.0	4.4 × 2.7	9.5	5.5 × 2.9	12.9	6.2 × 3.7	18.0
Thermal dose map area	5:00 min	3.4 × 1.3	3.6	4.4 × 1.6	5.6	4.5 × 2.2	7.9	5.9 × 2.9	13.2
	7:30 min	3.8 × 1.2	3.6	4.5 × 1.6	5.9	5.5 × 2.5	10.7	5.7 × 3.4	15.1
	10:00 min	4.0 × 1.4	4.5	4.5 × 1.9	6.8	5.4 × 2.5	10.7	6.3 × 3.1	15.5
	15:00 min	3.6 × 1.7	4.9	4.9 × 2.5	9.6	5.5 × 3.0	13.1	6.4 × 3.7	18.8

The ANOVA evaluation demonstrated a significant overall model effect of time and temperature on the necrosis area (*p* < 0.001) indicating that the model explains a substantial portion of the variance in necrosis area. The effects of temperature and time on necrosis area (in post-hoc analysis) were significant (*p* < 0.001 and *p* = 0.016, respectively).

The **results of the MRI thermometry** evaluation are provided in Table 1 (for temperature maps and for thermal-dose maps). The visualized lesion areas in the temperature maps ranged from 3.1 to 18.0 cm². The areas in the thermal-dose maps ranged from 3.6 to 18.8 cm². All measured lesion areas showed again increasing values for higher temperatures and longer MWA durations (Fig. 2, middle and right column).

**Fig. 3:**
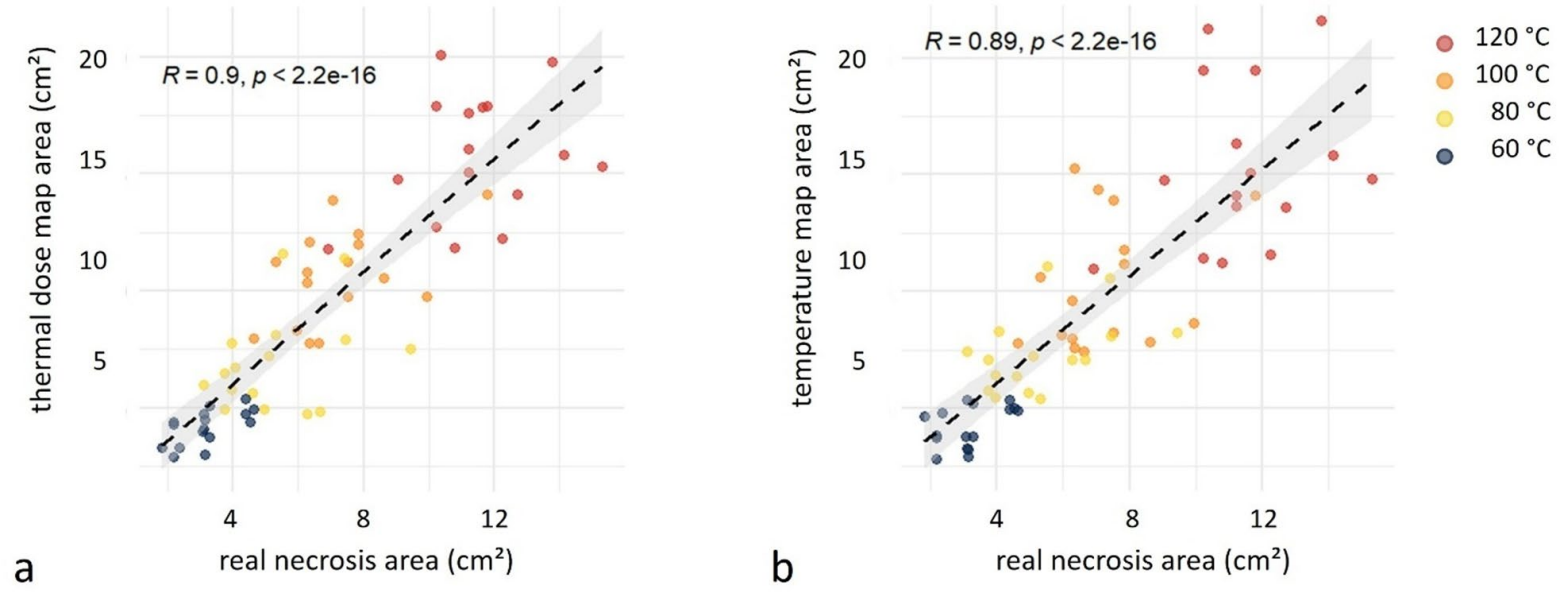
Correlation between necrosis area and thermometric map. Scatter plots illustrating the correlation between the measured area of real necrosis and the estimated ablation area derived from thermal dose maps (**a**) and temperature maps (**b**), expressed in cm². Data points are color-coded by temperature category (60 °C in dark blue, 80 °C in yellow, 100 °C in orange, and 120 °C in red). Each temperature category comprises 16 measurements (4 durations × 2 repetitions × 2 readers). The dashed black lines represent linear regression fits, with shaded bands indicating the 95% confidence intervals. Spearmann correlation coefficients (R) and corresponding p-values are shown in each panel.

The agreement between the MRI-based evaluation and the corresponding areas in the liver sections is illustrated in correlation scatter plots (Fig. 3) and Bland-Altman plots (**Supplementary Fig. ****S1**).

Correlation analyses further supported these findings, revealing a positive correlation between real necrosis and temperature map (correlation coefficient *R* = 0.89, *p* < 0.001). Similarly, a correlation was observed between real necrosis and thermal dose map (*R* = 0.90, *p* < 0.001).

When analyzing correlations by target temperature, correlations were found higher at low temperatures (below 100 °C) than high temperatures. At 60 °C, significant correlations were found with both maps (temperature: *R* = 0.702, *p* = 0.002; thermal dose: *R* = 0.655, *p* = 0.006). Similarly, at 80 °C, significant correlations emerged for the temperature map (*R* = 0.648, *p* = 0.007) and the thermal dose map (*R* = 0.766, *p* < 0.001). At 100 °C, no significant correlations were observed (temperature map: *R* = 0.421, *p* = 0.104; thermal dose map: *R* = 0.091, *p* = 0.738). The real necrosis area correlated with the temperature map at 120 °C (*R* = 0.524, *p* = 0.037), but not with the thermal dose map (*R* = 0.456, *p* = 0.076).

Finally, we performed NADH staining and H&E staining on frozen tissue samples to analyze differences in necrosis between 80 °C and 120 °C (**Supplementary Fig. S2**). These analyses showed no significant differences in the extent of necrosis or vital cell and enzymatic activities between the two temperature conditions.

### Image quality of thermometry maps

The Likert scores describing the **quality of the MRI maps** ranged from 1 (lowest quality) to 4 (out of 5, see **Supplementary Table ****S1**). The best quality maps, as reflected by Likert scores, were associated with lower temperatures. For example, at 60 °C for 15 min, the Likert score reached a maximum of 4 for the thermal dose maps, while at 120 °C, the Likert scores were generally lower, particularly for shorter durations (e.g., a score of 1.00 at 7.5 min). Overall, there is an inverse relationship between temperature and map quality, with lower temperatures resulting in better visualization.

**Fig. 4:**
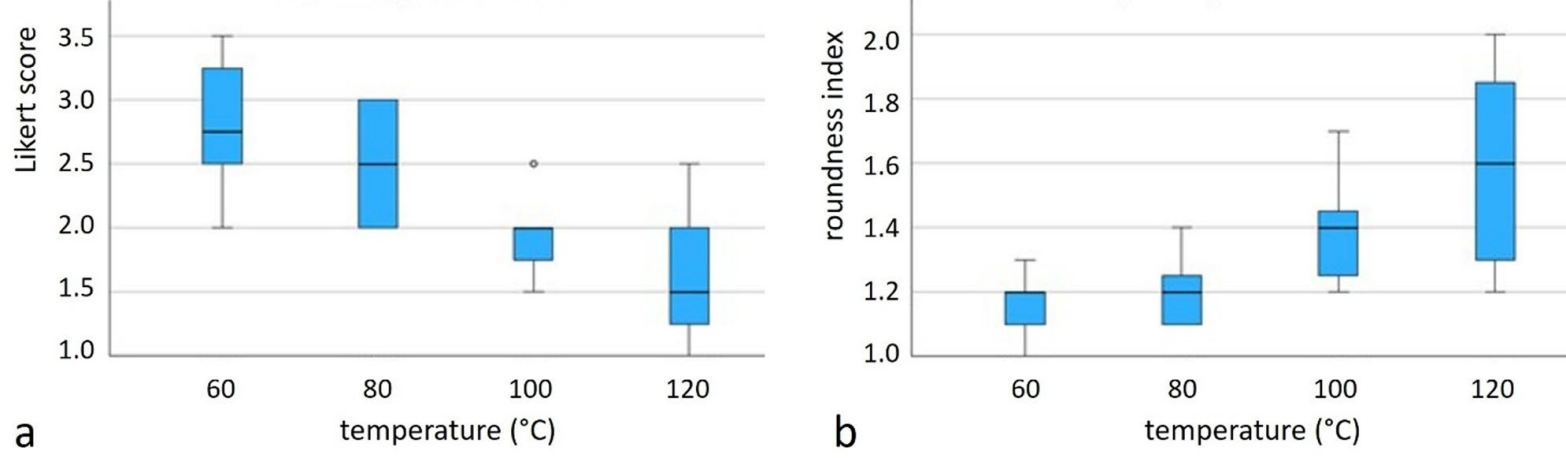
(**a**) Boxplots of Likert scores (image quality assessment, higher scores indicate better image quality) and (**b**) of roundness index (lower values indicate better image quality) stratified by target temperatures.

The Kruskal-Wallis analysis of the image quality revealed a significant difference in Likert scores across the four temperature groups (*p* < 0.001). Post hoc comparisons using the Mann-Whitney U test confirmed these findings. Significant differences were found between 120 °C and both 80 °C (*p* < 0.001) and 60 °C (*p* < 0.001), as well as between 100 °C and both 80 °C (*p* = 0.004) and 60 °C (*p* < 0.001). No significant difference was observed between 120 °C and 100 °C (*p* = 0.111) or between 80 °C and 60 °C (*p* = 0.400). These results highlight a progressive and significant deterioration of image quality with increasing temperature. The boxplot illustrating the distribution of Likert scores across temperature groups is presented in Fig. 4a.

The reconstructed shape of the temperature field, evaluated by the RI, provides further insight into the geometric quality of MRI thermometry (Fig. 4b). The RI ranged from 1.05 to 1.75, with a mean of 1.33. Lesions treated at 120 °C had a significantly higher (worse) RI (range 1.50–1.75) compared to lower temperature settings, indicating more irregular lesion shapes. Specifically, the 15:00-min group at 120 °C exhibited the worst RI of 1.75, while lower temperatures like 60 °C at 5:00 min had more regular shapes (index 1.05) (**Supplementary Table S2**).

The inter-reader agreement was excellent across all evaluated parameters, with ICCs for average measurements of 0.998 for thermal dose maps, 0.997 for temperature maps, 0.981 for the Likert scale, and 0.921 for the RI; detailed results are provided in **Supplementary Table S3**.

## Discussion

This study provides a controlled, preclinical evaluation of how target temperature and ablation duration influence the stability and interpretability of PRFS-based MR thermometry during microwave ablation, addressing a practical limitation of MR-guided procedures under standardized experimental conditions and aiming to identify strategies to optimize imaging reliability while maintaining sufficient ablation volumes^18,36,37^.

Our main findings demonstrate that higher target temperatures yield significantly larger ablation zones, yet concurrently lead to a marked deterioration in the quality of MRI thermometry maps. In contrast, image quality of MRI thermometry improved with decreasing temperatures. However, the resulting reduction in lesion size could not be fully compensated by merely extending the ablation duration.

Importantly, although improved thermometry stability and map interpretability were observed at lower temperatures under the investigated conditions, the primary objective of ablation remains safe and complete tissue destruction. Therefore, improved thermometry performance should not be interpreted as an argument for insufficient heating at the expense of ablation efficacy. Higher target temperatures, despite being more prone to susceptibility-related artifacts, may be necessary to achieve complete necrosis. Previous studies have explored MWA under MRI guidance from clinical and technical perspectives including approaches to artifact reduction. Recent advances in MRI technology have enhanced the efficacy and precision of MR-guided MWA. In particular, Ozenne et al. (2024) as well as Kim et al. (2024) highlight novel developments in motion-robust MR thermometry, which are crucial for improving the accuracy of thermal monitoring during ablation procedures. These advancements contribute to mitigate the effects of patient movement, ensuring more reliable thermometric data and better overall procedural outcomes^26,38^.

In a more recent study, Öcal et al. (2024) demonstrated that thermal dose predictions obtained via PRF-based real-time MR thermometry exhibit a correlation with the ablation zone delineated on imaging performed on the first post-interventional day. The visual assessment confirmed a high degree of concordance between the predicted thermal dose volumes and the actual ablation volume. Additionally, they noted that real-time visualization of inadequate ablation margins could help reduce local recurrence rates by allowing for immediate re-ablation of lesions during the same procedure^39^. However, in 18% of the analyzed cases MR thermometry quality was insufficient for reliable analysis, with heating-induced artifacts among the primary causes. These findings underline the necessity of further technical improvements to facilitate the routine clinical implementation of this method.

Assessing the influence of temperature and ablation time on necrosis area, we found that temperature exerted a more substantial impact on the necrosis area than ablation time. This highlights the role of temperature in determining the extent of tissue necrosis and suggests that optimizing temperature settings is more effective than prolonging ablation time. A significant correlation was observed between the temperature-derived ablation areas, obtained from temperature maps and thermal dose maps, and the actual necrosis area. This correlation was stronger at lower temperature settings, suggesting that MR thermometry at lower temperatures provide a more reliable temperature change maps with fewer susceptibility artifacts potentially due to gas formation through tissue vaporization. This finding is consistent with the hypothesis that at higher temperature, tissue phase changes (from water to vapor) induce susceptibility changes affecting the PRFS quantification^28^.

Our results about MRI image quality indicated that the thermometric maps improved at lower temperatures, with significant differences observed between higher and lower temperature settings (Fig. 4, Suppl. Tab. S1). The use of this scoring system provides a nuanced view of how artifacts and morphological features of the ablation zone impact the overall interpretability and diagnostic value of MRI thermometric maps.

To corroborate robustness of our findings, we evaluated the inter-reader agreement to assess the reliability of the MRI thermometric maps. The analyses demonstrated excellent concordance between independent readers, indicating that the maps provide consistent and reproducible results across evaluators. Such level of agreement is crucial for clinical applications, where reliable interpretation of thermometric data is essential for accurate treatment planning.

Based on our results, the most favorable compromise between achieving a sufficiently large necrotic area and maintaining high-quality MRI thermometry quality was observed at a target temperature of 80 °C for 15 min with the device used. At this setting, imaging quality was optimal and did not compromise the necrotic area, in terms of size and efficiency, as confirmed by histopathological staining. However, the obtained lesion size at 80 °C remains smaller compared to 120 °C, which may limit the effectiveness of treatment in larger targets. The 80 °C at 15 min protocol, identified as the best compromise between map quality and necrosis size, could serve as a calibration reference for future in vivo implementations and algorithmic correction strategies.

Furthermore, our findings suggest that a two-level approach may be advantageous when larger ablation volumes are required: First, an ablation of 80 °C is performed to visualize the thermal dose distribution around the MWA needle without strong artifacts, thereby enabling verification of correct ablation placement and allowing for applicator re-positioning if necessary. In a second step, once a homogeneous ablation area is observed, the target temperature can be increased to 120 °C to enlarge the ablation volume accepting a potentially reduction in MRI thermometry quality.

We acknowledge that MR thermometry performance is highly dependent on the specific sequence design, imaging parameters, hardware configuration, and tissue properties. Accordingly, the proposed two-level approach is proposed only for the investigated ex vivo bovine liver model and acquisition protocol, and should not be generalized to other tissues, systems, or field strengths without further validation.

The observed optimal thermometry performance at 60 °C with prolonged ablation duration should not be interpreted as a fundamental thermal or temporal threshold. Rather, it represents an empirical balance between sufficient thermal dose delivery and preservation of PRFS phase stability under the specific experimental conditions of this study. Lower target temperatures reduce tissue vaporization, gas formation, and abrupt susceptibility changes, thereby improving phase stability and temperature map interpretability. Prolonged heating partially compensates for the reduced peak temperature in terms of cumulative thermal dose, while avoiding the severe artifacts observed at higher temperatures.

Accordingly, the identified optimal temperature–time combination should be interpreted as condition-specific and may not translate directly to different tissues, perfusion states, hardware configurations, or thermometry sequences.

The ex vivo bovine liver model was intentionally selected to enable controlled optimization of MR thermometry parameters under static conditions, minimizing confounding factors such as motion, perfusion, and physiological variability. This stepwise approach reflects commonly adopted MR thermometry development workflows, including vendor-recommended validation strategies, in which protocol stability is first assessed ex vivo prior to in vivo translation.

This study has several limitations. First, the experiments were conducted ex vivo with a limited number of samples, which does not fully replicate the biological variability and tissue properties found in vivo conditions.

Additionally, artifacts related to respiratory motion, which can significantly impact the accuracy of PRFS-based MR thermometry, were intentionally not accounted for in the present experimental setting, in order to isolate heating- and susceptibility-related instabilities under static conditions. In clinical practice, respiratory motion is commonly addressed using motion mitigation strategies such as respiratory triggering or gating, which have been shown to enable quasi–motion-free PRFS thermometry during microwave ablation^39^.

Furthermore, the absence of tissue perfusion in ex vivo models represents an important limitation, as perfusion in vivo acts as a heat sink and strongly influences heat distribution, cooling effects, and the size and shape of the ablation zone. This experimental choice was intentional, reflecting a stepwise validation strategy commonly adopted in MR-guided ablation, in which protocol optimization and thermometry stability are first assessed under controlled, non-perfused conditions before translation to in vivo settings.

This study did not include classical MRI physics metrics such as temperature-to-noise ratio or absolute temperature resolution, which are essential for full metrological validation of PRFS thermometry. This choice reflects the comparative aim of the study, which was designed to evaluate relative thermometry stability and visualization quality across different heating strategies rather than absolute temperature accuracy.

Although an image-based drift correction was applied, residual *B*_0_ drift-related errors cannot be completely excluded during prolonged ablations.

Finally, it should be noted that thermometry performance is influenced by the specific implementation, reconstruction pipeline, and vendor-dependent processing. Accordingly, the present results should not be assumed to be directly transferable to other MR thermometry systems without dedicated validation.

## Conclusion

Our study provides valuable insights into the application of MR thermometry for monitoring MWA. Lower target temperatures were associated with better MRI thermometry quality in this ex vivo study, providing more reliable visualization of ablation zones. However, this advantage comes at the expense of smaller lesion sizes potentially limiting therapeutic efficacy and requiring careful consideration in clinical decision-making. The findings highlight the potential of MR thermometry to enable precise visualization of ablation procedures, but also reveal the limitations of MRI thermometry quality at higher target temperatures ≥ 100 °C. To visualize thermal effects and to allow for more accurate assessment of ablation margins, the use of lower target temperatures appears advisable whenever possible. Future research should address the limitations identified, particularly by evaluating in vivo conditions and systematically accounting for the effects of respiratory motion and tissue perfusion. Such efforts will be essential to further refine and optimize MR-guided MWA techniques.

## Supplementary Information

Below is the link to the electronic supplementary material.

Supplementary Material 1

## Data Availability

All data being analyzed as part of this study are included in this manuscript and the supplementary materials. Further inquiries can be sent to the corresponding author.

## Abbrevations

HCC: Hepatocellular carcinoma
MWA: Microwave ablation
EASL: European association for the study of liver
BCLC: Barcelona clinic liver cancer
RFA: Radiofrequency ablation
US: Ultrasound
CT: Computed tomography
MRI: Magnetic resonance imaging
PRFS: Proton resonance frequency shift
EPI: Echo planar imaging
CEM: Comulative equivalent minutes
H&E: Hematoxilin and eosin
ICC: Intraclass-correlation coefficient
ANOVA: Analysis of variance
