# Supplementary material for: Quantitative ex vivo assessment of target temperature and ablation duration for protocol optimization of microwave ablation procedures with mr thermometry
Source: Sci Rep. 2026 Mar 3;16:8153. doi: 10.1038/s41598-026-41656-3 (PMC12960678; doi:10.1038/s41598-026-41656-3)
Supplement: Supplementary file 1 — Supplementary Material 1 [file 41598_2026_41656_MOESM1_ESM.docx]

**Improving MR thermometry for microwave ablations: Quantitative assessment of target temperature and ablation duration**

Supplementary Materials

# Supplementary Methods

## Histological sample analysis

Representative tissues from different ablation temperatures and control tissues were obtained and sectioned at 8 µm thickness using a cryostat (Leica Biosystem cryostat CM1510, Wetzlar, Germany) for histological analysis. Cryosections were stained with a solution of reduced NADH (nicotinamide adenine dinucleotide) and NBTC (nitro blue tetrazolium chloride) to assess cellular metabolic activity and differentiate between vital and non-vital cells, as previously described by Neumann et al. (1991) [30, 31]. Additionally, consecutive frozen sections from the same specimens were stained with hematoxylin and eosin (H&E) to evaluate tissue maintenance. Slides were scanned using a digital slide scanner (Pannoramic Scan II, 3D Histech Ltd., Budapest, Hungary), and photographs were captured using SlideViewer 2.8 software (3D Histech Ltd., Budapest, Hungary) at 0.3× magnification.

# Supplementary Tables

**Supplementary Table S1:** Likert score results of MRI thermometry maps

|  | **MWA duration** | **60°C** | **80°C** | **100°C** | **120°C** |
| --- | --- | --- | --- | --- | --- |
| **Temperature maps** | 5:00 min | 2.00 | 2.00 | 2.00 | 1.00 |
|  | 7:30 min | 3.00 | 2.50 | 2.00 | 1.00 |
|  | 10:00 min | 2.00 | 2.00 | 1.00 | 2.00 |
|  | 15:00 min | 3.00 | 3.00 | 2.00 | 2.00 |
|  |  |  |  |  |  |
| **Thermal dose maps** | 5:00 min | 3.00 | 2.25 | 2.00 | 2.00 |
|  | 7:30 min | 3.00 | 2.50 | 2.50 | 1.00 |
|  | 10:00 min | 2.25 | 3.00 | 2.00 | 2.00 |
|  | 15:00 min | 4.00 | 3.00 | 2.00 | 1.50 |
|  |  |  |  |  |  |
| **Overall** | 5:00 min | 2.50 | 2.12 | 2.00 | 1.50 |
|  | 7:30 min | 3.00 | 2.50 | 2.25 | 1.00 |
|  | 10:00 min | 2.12 | 2.50 | 1.50 | 2.00 |
|  | 15:00 min | 3.50 | 3.00 | 2.00 | 1.75 |

**Supplementary Table S2.** Roundness index of lesions on thermometry maps

|  | **60°C** | **80°C** | **100°C** | **120°C** |
| --- | --- | --- | --- | --- |
| **5:00 min** | 1.05 | 1.20 | 1.45 | 1.50 |
| **7:30 min** | 1.15 | 1.12 | 1.35 | 1.55 |
| **10:00 min** | 1.20 | 1.20 | 1.35 | 1.50 |
| **15:00 min** | 1.17 | 1.25 | 1.43 | 1.75 |

(ratio of long and short axes of the lesion perpendicular to the needle on thermometry maps reformatted para-sagittally)

**Supplementary Table S3.** Intraclass correlation between the two readers

| **Intraclass correlation (ICC) values** | | | | | |
| --- | --- | --- | --- | --- | --- |
|  | **Averaged measurements** | | **Single measurements** | | **ICC matrix** |
|  | **ICC coefficent (95%CI)** | **p-value** | **ICC coefficent (95%CI)** | **p-value** |  |
| **Thermal dose map** | 0.998 (0.997-0.999) | <0.001 | 0.997 (0.993-0.998) | <0.001 | 0.997 |
| **Temperature map** | 0.997 (0.993-0.998) | <0.001 | 0.993 (0.986-0.997) | <0.001 | 0.994 |
| **Likert scale** | 0.981 (0.962-0.991) | <0.001 | 0.963 (0.927-0.982) | <0.001 | 0.963 |
| **Roundness Index** | 0.921 (0.838-0.961) | <0.001 | 0.853 (0.731-0.926) | <0.001 | 0.853 |

(p-values calculated using two-way random effects model for absolute agreement)

# Supplementary Figures


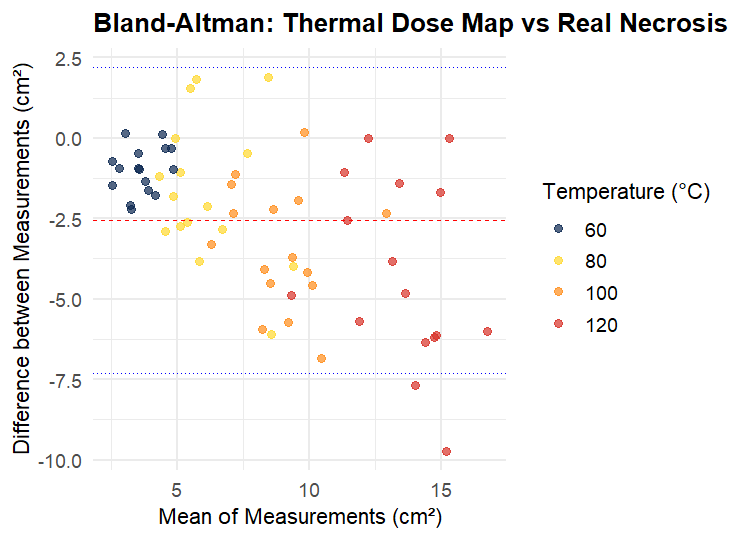

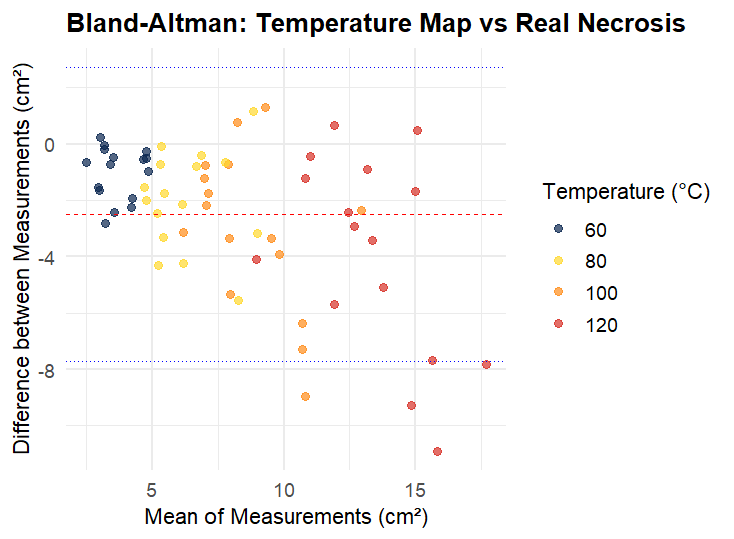


**Supplementary Figure S1.** Bland-Altman plots comparing MRI-derived maps with real necrosis area. (a) Thermal dose map vs real necrosis; (b) Temperature map vs real necrosis. Data points are color-coded by temperature category (60°C in red, 80°C in green, 100°C in blue, and 120°C in purple). Each temperature category comprises 16 measurements (4 MWA durations × 2 repetitions × 2 readers). The red dashed line indicates the mean bias, blue dotted lines represent the 95% limits of agreement.


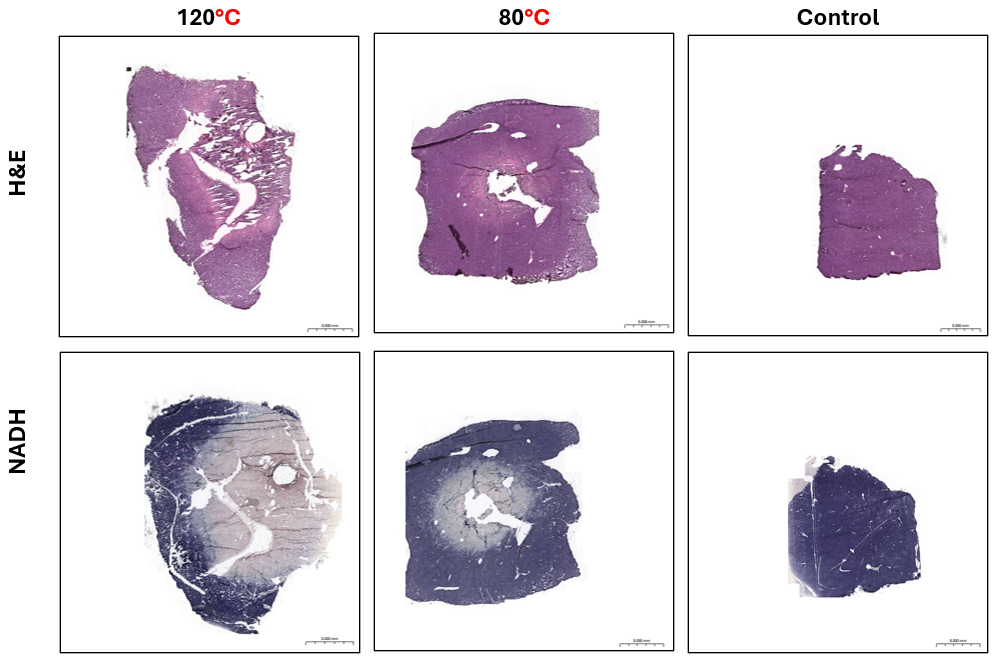


**120°C**

**80°C**

**Control**

**Supplementary Figure S2.** Representative H&E and NADH staining of ablation and control tissues (×0.3 magnification)
